# The Incidence of Mental Disorders Increases over Time in Patients with Cancer Pain: Data from a Retrospective Cohort Study

**DOI:** 10.1155/2021/5515629

**Published:** 2021-06-03

**Authors:** Michael Brinkers, Giselher Pfau, Anne-Marie Toepffer, Frank Meyer, Moritz A. Kretzschmar

**Affiliations:** ^1^Department of Anaesthesiology and Intensive Care Medicine, University Hospital Magdeburg, Magdeburg 39120, Germany; ^2^Augenzentrum Leiterstrasse, Magdeburg 39104, Germany; ^3^Department of General, Abdominal Vascular and Transplant Surgery, University Hospital Magdeburg, Magdeburg 39120, Germany

## Abstract

**Background:**

It is well known that cancer patients more seldom have a psychiatric disorder than noncancer patients with chronic pain. Conversely, earlier studies have suggested that, at the psychiatric level, long-term cancer survivors (LCSs) have more in common with noncancer patients affected by chronic pain.

**Materials and Methods:**

We investigated 89 cancer patients with acute pain (Acute Cancer Pain Patients, ACPPs) treated at a university outpatient chemotherapy department and compared these with 61 LCSs (living >5 yr after the first diagnosis) admitted by general practitioners for the treatment of noncancer pain. Upon administration, each patient was psychiatrically assessed by a liaison-psychiatrist conducting a semistructured interview. In a second step, we compared the LCS patients with hitherto treated noncancer patients suffering from chronic pain and ACPPs with data published by Derogatis in 1983.

**Results:**

In a comparison of LCSs with ACPPs, LCSs have more patients with brain organic disorders and more addictions. The largest cancer group within the LCSs is patients with urogenital (Uro) cancer (44.3%), while within the ACPPs, these are patients with cancer of the gastrointestinal (GI) tract (ACPP-GI, 57.2%). As far as the distribution of mental disorders is concerned, long-term cancer survivors show some similarities to noncancer patients. The data of ACPPs are similar to those of cancer patients, published by Derogatis. *Discussion*. The higher values of addiction and brain organic disorders, in particular, and the slight differences for psychic disorders in general of LCSs vs. ACPPs may result from the different cancer types and a longer survival time for urogenital tract cancer compared to GI cancer. In an additional examination, we compared patients with acute cancer of the GI tract (ACPP-GI, *n* = 50) with those of the urogenital tract (ACPP-Uro, *n* = 43). ACPP-Uro had the lowest percentage of patients with psychiatric disorders in general (ACPP-Uro 37.2%, ACPP-GI 50.0%, all LCSs 65.6%, and LCS-Uro 74.1%) and addiction, in particular (ACPP-Uro 2.3%, ACPP-GI 4.0%, and LCSs 13.1%).

**Conclusion:**

Cancer patients can develop a process of chronification with an increase in the prevalence of mental disorders. For urogenital cancer, an increase in the probability to develop mental disorders is a function of time.

## 1. Introduction

The term “Long-term Cancer Survivors (LCSs)” stands for patients who stay alive five years and more after the first diagnosis and subsequent therapy of malignant cancer lesions. According to the National Cancer Institute, a survivor is defined as “the one who remains alive and continues to function during and after overcoming a serious hardship or life-threatening disease. In cancer, a person is considered a survivor from the time of diagnosis until the end of life [1].”

In the USA, the number of cancer survivors registered in 2013 was estimated to range from 11.4 million [2] to 13.7 million [3] depending on the source of data. This number increased to 15.5 million in 2016 [4]. According to Irwin, two-thirds of these are LCS patients. In Germany, Arndt et al. [5] estimated about 3.2 million LCS patients [6].

Among numerous other aspects, LCS patients can suffer from persisting pain. LCSs with pain more often have financial problems compared with painless survivors [6] and are more frequently affected by depression [7]. Furthermore, such patients can develop a fear that they might be excluded from social life [8]. Indeed, they increasingly report problems related to social exclusion. They can have difficulties in accepting the disease, and they can suffer from reduced quality of life. This may lead to a lack of exercise and weight gain [9], two factors possibly related to pain and mental disorders. These patients fear recurrence, relationship difficulties, financial and existential problems, and problems returning to work. Strategies that allow them to cope with cancer even if it has been overcome suddenly take an important part in their life [10, 11]. Fear-avoidance beliefs and efforts as well as a possible lack of psychosocial support can, in turn, be associated with an increased rate of pain manifestation [12–14].

The fear that the same or another cancer might recur possibly explains the increased psychological distress in LCSs. According to D'Agostino et al. [15], the number of patients with leukemia, cancer of the central nerve system, and sarcomas, who are vulnerable to distress, is higher than that of patients with (other) solid tumors. As distress increases pain sensitivity, the design of pain therapy is important.

Mental disorders can also lead to added substance addictions. Wirz et al. [11] presumed that, in contrast to acute cancer patients, LCSs may also develop opioid addiction owing to the psychological factors mentioned above. According to the literature, treatment of LCS patients by exclusive administration of opioids is more critical than the same treatment given to cancer patients [16], especially taking into account side effects and increased administration of psychotropic drugs [17]. Therefore, psychotherapeutic measures (to reduce distress) are considered more important [18, 19]. Cancer patients subjected to active strategies to cope with their disease more rarely report chronic pain.

Under the condition that patients were interviewed using a structured or semistructured interview to obtain a diagnosis according to the diagnostic manual (DSM III-V/ICD-10), the following distribution can be found: on the one hand, acute tumor patients 15–50% [20–24] and LCSs 56% [25], and, on the other hand, chronic pain patients without cancer around 70% [26–29].

Following this succession of numbers, one can presume that the incidence of psychiatric disorders increases over time from the initial cancer diagnosis.

To our knowledge, there are no studies in which a single psychiatrist (and a single team) has diagnosed and treated acute and long-term cancer patients as well as noncancer patients with chronic pain and has compared the courses of treatment.

We attempt to answer the question if this progression over time in psychiatric diagnoses can be verified by looking at

1. the difference between the spectrum of psychiatric diagnoses of LCS patients and that of patients with chronic pain
2. the difference between ACPPs and the data collected by Derogatis et al. [22]
3. if there is evidence of an increase in psychiatric diagnosis over time in cancer patients who have survived for more than 5 years compared to patients admitted to treatment for acute cancer pain

## 2. Patients and Methods

### 2.1. Investigation Procedure

In this retrospective cohort study, a Consultant-Liaison (CL)—a psychiatrist working at the Magdeburg Outpatient Pain clinic—has been collecting data on all admitted pain patients' psychiatric findings and social medical history since 2001. In cancer patients, social history data and psychological findings (analogous to primary pain patients without underlying cancer) were collected in addition to pain-associated aspects between 2012 and 2016.

**Figure 1:**
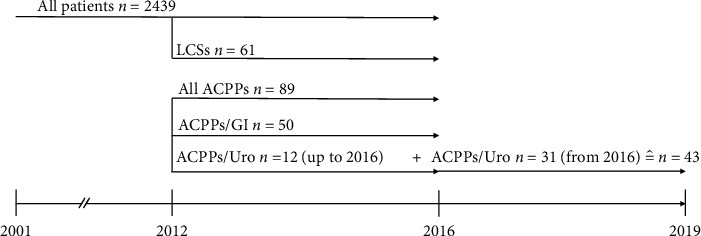
Timeline of patient inclusion; LCSs: long cancer survivors; ACPPs: acute cancer pain patients; GI: gastrointestinal; and Uro: urology.

The control group consisted of all patients treated at the pain clinic up to the end of 2016 and whose medical history included treatment of previous cancer with a minimum 5-yr cancer-free survival period (LCSs) (Figure 1).

The parameters investigated were as follows:

- Education, family status, and profession
- Localization of cancer and pain
- Psychiatric diagnoses according to ICD-10

No patients were omitted due to missing data.

### 2.2. Patients

The cancer patients were recruited from the two chemotherapy divisions of the university hospital as well as from various consulting university hospital departments. The control group of LCS patients consisted of patients referred to us by general practitioners specifically for the treatment of noncancer pain. Upon admission at the Pain Management Clinic, the oncological treatment had been completed.

#### 2.2.1. Exclusion Criteria

Cases of severe dementia or other disorders that did not allow for an orderly diagnosis on the psychiatric level (including patients not speaking the German language) were excluded, as were new admissions of LCS patients due to pain caused by a newly formed tumor.

### 2.3. Acquisition of Psychiatric Data

The same questionnaire based on the Association for Methodology and Documentation in Psychiatry (AMDP) was used for all patients. The psychiatric diagnosis was made by combining the psychiatric findings, family and social history, and the criteria based on ICD-10. The investigating CL-psychiatrist (M.B.), a medical specialist in psychiatry since 2000, joined the palliative ward in 2001 and has been working at that department as a CL-psychiatrist ever since. He interrogates each patient admitted to the palliative ward in a personal interview to acquire psychiatric data.

### 2.4. Design of the Comparison Groups

The comparison group for the long-term cancer survivors (LCSs) consisted all patients who were not registered as acute cancer pain patients (ACPPs) in the palliative ward or the pain management clinic and who were admitted and treated at the outpatient clinic up to the end of 2016 (*n* = 2439).

The data of a previous U.S. American study conducted by Derogatis et al. [22] served as the historical comparison group of patients admitted to a pain management clinic because of acute cancer pain. These data were published in the Handbook of Psychooncology by Holland and Rowland ([30], p. 274). The DSM-III-diagnoses described by them were “translated” into the ICD-10 code as follows (in brackets).

Organic (F0), addiction (F1), schizophrenia (F2), major affective disorders (F3), adjustment disorders + anxiety disorders (F4), psychosomatics (F5), personal disorders (F6), intellectual capacity (F7), and psychiatric diagnosis absent (absent = inconspicuous).

### 2.5. Statistics

The data were evaluated using IBM SPSS Statistics, version 24 (Chicago/IL, U.S.A.).

#### 2.5.1. Descriptive Statistical Analyses

Frequencies were calculated for qualitative characteristics and indicated as absolute percentages. For quantitative characteristics, position and scattering parameters were determined (median and standard deviation).

For comparison, these descriptive analyses were carried out for LCS patients and chronic pain patients.

#### 2.5.2. Statistical Tests

The comparison of qualitative variables between these two patient groups was made by contingency table analyses using Pearson's chi-squared test as appropriate.

Quantitative variables (due to deviations from the normal distribution assumptions) were compared by nonparametric Mann–Whitney's U test between the patient groups. In all statistical tests, an error probability of alpha <0.05 was considered significant.

### 2.6. Statement

The study was performed according to the principles of the Declaration of Helsinki of the World's Association of Physicians for Biomedical Research from 1964 and its further amendments as well as according to the policy of the institutional ethics committee.

## 3. Results

### 3.1. General Data

Table 1 gives an overview of the demographic and sociopsychological data. In LCS patients, there were a larger proportion of women, while in the ACPP group, there were more men.

  Education: LCS patients have completed the 8^th^ grade of school in a higher percentage and, compared to the ACPP group, the 10^th^ grade in a lower percentage (chi-squared test: *p*=0.046)
  Family status: comparing LCSs and ACPPs, there were no significant differences (chi-squared test, *p*=0.821)
  Profession: in its distribution among groups, no significant difference (chi-squared test, *p*=0.211)
  Psychiatric distress: there were no significant differences (LCSs: 57.0 ± 11.1, ACPPs: 55.9 ± 11.5; Mann–Whitney's U test, *p*=0.543)

Age was not significantly different (LCSs: 64.1 ± 12.7, ACPPs: 63.7 ± 10.9; Mann–Whitney's U test, *p*=0.848).

However, there were significant differences (as expected) comparing the time from the first diagnosis of cancer and patients' admission to the pain clinic (LCSs: 10.6 ± 4.9, ACPPs: 2.2 ± 4.0; Mann–Whitney's U test, *p* < 0.001).

In LCS patients, the cancer was more frequently localized in the urogenital tract and less frequently in the gastrointestinal (GI) tract (chi-squared test, *p* < 0.001), and vice versa in ACPPs (Table 2).

### 3.2. Distribution of F-Diagnoses

Regarding F-diagnosis, LCS patients (dark) had significantly more brain organic diagnoses (F0) and more addictions (F1) (Pearson's chi-squared test, *p* 0.007).

## 4. Discussion

This study is the first to our knowledge to directly compare oncologically treated cancer patients with acute pain (ACPPs) and Long Cancer Survivors (LCSs) whose oncological treatment has been completed.

The distribution of psychiatric disorders of LCSs is equal to that of all noncancer patients of the pain management clinic [31]. The diagnosis distribution of ACPPs is not different from that reported by Derogatis et al. [22, 30].

**Figure 2:**
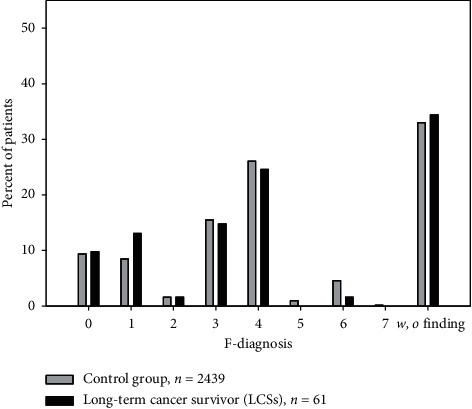
Distribution of psychiatric diagnoses (in percent) comparing LCS patients (with long-lasting pain) and the control group. Light bar: control group = the whole group of all chronic pain patients admitted to the pain management clinic (*n* = 2,439; dark bar: LCSs, *n* = 61); *w*/*o* finding: psychiatrically inconspicuous regarding formal criteria. The distribution of both groups is not significantly different (chi-square test, *p*=0.847).

Compared with ACPPs, the LCS group (dark, Figure 2) has more patients with brain organic disorders and more patients with substance addiction [11]. This might be due to two reasons: the course of time and incidence of psychiatric diagnoses in different cancer types.

### 4.1. Course of Time

At the time of admission, the patients of both groups (LCSs and ACPPs) are at the same age (LCSs: 64.1 ± 12.7 yr; acute: 63.7 ± 10.9 yr. The time between the first diagnosis of cancer and admission to the pain management clinic is considerably longer for LCS patients than for ACPPs (10.6 ± 4.9 vs. 2.2 ± 4.0 yr). At the end of the therapy, only 15 patients were LCSs according to the definition (minimum 5 years, up to 10 years), while the remaining 46 patients had survived more than10 years (11 to 41 years).

In both groups (LCSs and ACPPs) one type of cancer predominates, respectively: in LCSs, it is the urological tract (44.3%), and in ACPPs, it is the GI tract (56.2%). There is a different distribution in the proportion of outpatients. Out of the 55 patients with GI cancer, 50 (90.9%) were referred from chemotherapy divisions outpatient clinics (acute), while only 12/39 (30.8%) of the urogenital cancer patients came from outpatient clinics. The time between first diagnosis and admission are correspondingly different (GI cancer: 1.55 ± 2.67 yr; urological cancer: 11.72 ± 10.88 yr).

Therefore, the higher frequency of mental disorders in the LCSs can be explained by the higher survival rate (patients with cancer of the urogenital tract in comparison to those with cancer of the GI tract). According to the CONCORD-2 study [32] conducted in 2004/2005, the 5-yr survival rate in patients with gastric cancer is 31.6%, while patients with cervical cancer have a 5-yr survival rate of 64.9%, prostate cancer, even 91.2%.

There might exist a psychiatric “factor” of a subsyndromal psychiatric (reactive) alteration already existing in urogenital cancer and noticed by the patients themselves at first which, more typical for or associated with the long survival time, might develop as a clinical manifestation [33]. Therefore, LCS patients fear being confronted with recurrences (patients successfully treated for a primary cancer are susceptible to secondary cancer occurring after years [34]), relationship problems, financial and existential aspects, and problems returning to work. This psychiatric factor only becomes more meaningful over the years. Doege et al. [35] verified the possibility of existing but unquestioned complaints in LCSs only detected by changing the list of questions.

### 4.2. Incidence of Psychiatric Diagnoses in Different Cancer Types

**Figure 3:**
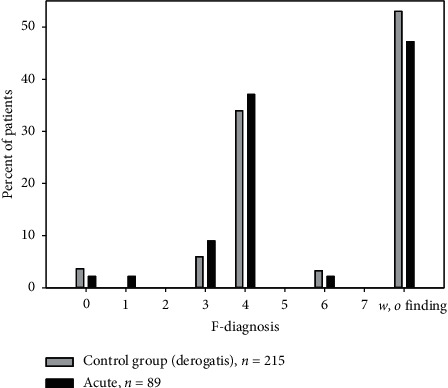
Distribution of psychiatric diagnoses (in percent) comparing ACPPs (acute cancer pain patients) and the historic control group (Derogatis et al. [22]). Light bar: control group = psychiatric diagnosis in cancer patients according to Derogatis et al. [22] (*n* = 215); dark bar: acute (ACPPs) (*n* = 89); *w*/*o* finding: psychiatrically inconspicuous. F3-depressions in acute cancer patients=9%; according to Derogatis et al. [22], 6.0%. The distribution of both groups is not significantly different (chi-square test, *p*=0.232).

**Figure 4:**
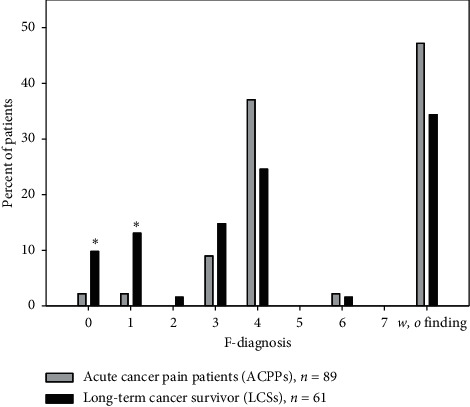
Distribution of psychiatric diagnoses (in percent) in LCS patients (dark bars) vs. ACPPs (light bars). ^*∗*^*p*=0.007, *p* ≤ 0.001.

The number of mental disorders found in long-term cancer survivors (LCSs) in this study is not higher than in ACPPs. The overall incidence of mental disorders in the LCS group was also higher than that previously reported (67% vs. 56%) [20]. ACPPs as patients out of Derogatis' study are mentally not affected by approximately 50% (53.0%, 47.2%, in our patients, Figure 3), while cancer patients with chronic pain (LCSs) and the noncancer patients of our pain clinic (34.4% vs. 33.0%, Figure 4) are only affected by one-third.

Among the 150 patients of our study (LCSs and ACPPs), 26 (47.3%) of the 55 patients with cancer of the GI tract (mostly ACPPs) suffered from a mental disorder vs. 27 of 39 patients (69.2%) with cancer of the urogenital tract (most of them were LCSs). This means that patients with cancer of the urogenital tract are more “susceptible” to mental disorders. This assumption is in accordance with Park et al. [36] who investigated the ten most important cancer types. Especially regarding depressions, the highest rate was found in lung cancer (11.0%) followed by tumor lesions of the urogenital tract (8.8–9.1), while there is a prevalence of 6.5–7.7% for cancers of the GI tract [36].

### 4.3. There Are Critical Objections to These Data

Although according to Park et al. [36], there is a different prevalence of F-3 depression among the various cancer types, this is not evident in the patients as presented (ACPP-GI: *n* = 50, F3 = 10.0%; LCS-Uro: *n* = 27, 11.0%), and in the total groups for LCSs (*n* = 61) and ACPPs (*n* = 89) for depression of F3 as well as for F4 diagnosis, there are only slight (nonsignificant) differences comparing chemotherapy patients from outpatient clinics and chronic noncancer patients.

Nevertheless, the tendency towards a higher number of mental diseases in LCS patients with cancers of the urogenital tract might be due to a mixture of susceptibility to psychiatric diagnoses and a higher survival rate.

In an attempt to answer the question of whether the higher incidence of psychiatric diagnoses in the LCS group is due to the specific cancer or due to the different long-term clinical courses, 50 ACPPs with GI cancer from the chemotherapy outpatient clinics (the subgroup of the ACPPs) were compared with 43 ACPP patients with urogenital cancer (12 patients from the initial timeframe of 2012 to 2016 and 31 further patients from 2017–2019) from the urology outpatient department in an additional study [37].

If the higher number of mental disorders in long-term urogenital cancer survivors, as shown in Figure 2, was a function of the localization of cancer and not of time, the patients with acute urogenital cancer would have more mental disorders when compared to acute cancer pain patients with GI-tract cancer. Yet, when looking at acute pain patients with urogenital cancer [37], these have a low incidence of mental disorders (37.2% compared to 50.0% in acute GI cancer and 74.1% in LCS-Uro).

### 4.4. Limitations

Due to the different numbers of patients with the various types of cancer, there was only a correlation of the number of psychological findings as found over time in patients with urogenital cancer.

The findings of Park et al. [36] that patients with urogenital tract cancer lesions have more depressions than patients with cancer lesions of the GI tract cannot be confirmed here due to the limited number of patients (depression in ACPP-Uro, *n* = 1).

## 5. Conclusions and Consequences for Clinical Practice

- Cancer patients can develop a process of chronification. An important feature is a significant increase in the prevalence of mental disorders, in particular, addiction.
- In patients with urogenital cancer, it could be shown that an increase in the probability to develop mental disorders is a function of time. Patients with a rather long history of manifest cancer must be repeatedly examined for mental disorders. When taking urogenital cancer as an example, the number of concomitant mental disorders increases over time.
- Psychic disorders can only be diagnosed by a psychologist or a physician experienced in the psyche by means of a personal interview. Screening alone is not sufficient. The diagnosis of mental disorders is a prerequisite for the initiation of an appropriate therapy as a supplement to pain therapy and/or psycho-oncological measures.

## Figures and Tables

**Table 1:** Demographic and sociopsychological data of patients with cancer history ≥5 y (LCSs) and acute cancer pain patients (ACPPs).

Parameter	LCSs (*n*)	LCSs (%)	ACPPs (*n*)	ACPPs (%)
Gender (f/m)	36/25	59/41^*∗*^	42/47	47.2/52.8^*∗*^
Age (years)	64.1 (12.7)		63.7 (10.9)	

*Education*				
No data	2	3.3	0	0.0
Up to the 8^th^ grade	31	50.8^*∗*^	32	36.0^*∗*^
10^th^ grade	20	32.8^*∗*^	43	48.3^*∗*^
School leaving examination	7	11.5	8	9.0
Studies/degree course	1	1.6	6	6.7

*Family status*				
No data	2	3.3	1	1.1
Unmarried	3	4.9	3	3.4
Married	42	68.9	68	76.4
Divorced	6	9.8	8	9.0
Widowed	8	13.1	9	10.1

*Profession*				
No data	2	3.3	1	1.1
Freelancer	2	3.3	1	1.1
Executive employee	0	0.0	2	2.2
T.p.G.^#^	9	14.8	21	23.6
Office or sales department	6	9.8	6	6.7
Specialist worker	39	63.9	48	53.9
Worker with a.s.^##^	2	3.3	6	6.7
Unskilled worker	0	0.0	4	4.5
No profession	1	1.6	0	0.0

^#^Technical professional graduation; ^##^acquired skill; ^*∗*^*p* < 0.05.

**Table 2:** Cancer localization in patients with cancer history ≥5 y (LCSs) and acute cancer pain patients (ACPPs).

Parameter	LCSs, *n* = 61	LCSs (%)	ACPPs, *n* = 89	ACPPs (%)
Head and neck region	6	9.8	10	11.2
GI tract	5	8.2^*∗*^	50	56.2^*∗*^
Respiration tract	3	4.9	6	6.7
Breast	10	16.4^*∗*^	4	4.5^*∗*^
Urogenital tract	27	44.3^*∗*^	12	13.5^*∗*^
Lymphatic hematopoietic system	2	3.3	0	0.0
Muscle, skin, bone, and connective tissue	8	13.1	6	6.7
Other or more than 1 localization	0	0.0	1	1.1

^*∗*^ *p* < 0.05.

## Data Availability

Previously reported data were used to support this study and are available at PMID: 6823028, DOI: 10.1001/jama.249.6.751. This prior study is cited at relevant places within the text as reference [22]. The data used to support the findings of this study are available from the corresponding author upon request.
